# The Impact of Dosing Interval in a Novel Tandem Oral Dosing Strategy: Enhancing the Exposure of Low Solubility Drug Candidates in a Preclinical Setting

**DOI:** 10.1155/2011/528284

**Published:** 2011-01-12

**Authors:** Po-Chang Chiang, Sarah A. South, Steve P. Wene

**Affiliations:** Pfizer Global Research and Development, St. Louis Laboratories, 700 Chesterfield Parkway West, Chesterfield, MO 63017, USA

## Abstract

In drug discovery, time and resource constraints necessitate increasingly early decision making to accelerate or stop preclinical programs. Early discovery drug candidates may be potent inhibitors of new targets, but all too often exhibit poor pharmaceutical or pharmacokinetic properties that limit the *in vivo* exposure. Low solubility of a drug candidate often leads to poor oral bioavailability and poor dose linearity. This issue is more significant for efficacy and target safety studies where high drug exposures are desired. When solubility issues are confronted, enabling formulations are often required to improve the exposure. However, this approach often requires a substantial and lengthy investment to develop the formulation. Previously, we introduced a gastrointestinal (GI) transit time-based novel oral tandem dosing strategy that enhanced *in vivo* exposures in rats. In this study, a refined time interval versus dose theory was tested. The resulting *in vivo* exposures based on altering frequency and doses were compared, and significant impacts were found.

## 1. Introduction

In the pharmaceutical industry today, an increasing number of low solubility drug candidates are providing scientists with the challenge of reaching desired exposures *in vivo*. Novel technologies have been developed for both the clinical and preclinical drug delivery of poorly soluble molecules [1, 2]. The pharmaceutical industry has increasingly pushed towards a programmatic “fail fast/fail cheap” paradigm in an effort to reduce costs and allocate resources in an efficient manner [3]. For a research program, early assessment of the efficacy and safety is often dependent upon efficient drug administration to generate reliable *in vivo* results in animal models for a “go” or “no go” decision. However, early drug candidates often exhibit poor pharmacokinetic attributes and physicochemical properties, such as poor solubility, making *in vivo* activity assessment difficult due to low exposure. Formulation-based approaches to improve exposure of these compounds, such as the addition of organic co-solvents, cyclodextrin, or emulsions, are most commonly used. However, the above approaches may interfere with the pharmacodynamic readout of the *in vivo* model or may not be tolerated by the subjects if multiple dosing is required to reach sustained systemic levels [4, 5]. Such issues become more problematic when early lead candidates are used for target safety evaluation where no interference from the vehicle formulation is allowed. Delivery options such as the use of biomaterials and polymeric delivery systems have been developed to address these issues [6, 7]. However, these tools are often costly and require a large amount of drug which makes them more suitable to be applied in a drug development environment. Other delivery technologies such as nano- and microparticle drug delivery systems have been applied throughout the pharmaceutical industry. These systems have mainly focused on oral, intraperitoneal, intramuscular, or subcutaneous delivery [8–10]. Theoretically, particle size reduction only improves dissolution by increasing surface area as described by Noye-Whitney, and marginally improves solubility as describe by Oswald-Freundlich [11]. Unfortunately, these improvements often fall short to overcome the solubility limited absorption when the dose is increased. Frequently, when solubility limit absorption is encountered, researchers have no choice but to wait for a more suitable drug candidate which often results in delay and increased cost. In many cases, higher doses (i.e., 1000 to 2000 mg/kg) are used *in vivo* in a futile attempt to increase exposure. This only wastes time and drug without answering the critical questions. In some cases where the linear absorption range of a drug can be found, b.i.d. (twice a day, every 12 hours) or t.i.d. (three times a day, every 8 hours), doses are used to increase exposure in model animals. However, these approaches often require significant staffing investments (late night shifts) which are not welcome. Moreover, for a compound with high clearance, drug accumulation after b.i.d. or t.i.d. dosing will be less significant. Such a dosing regimen will result in higher exposure (AUC) but with no *C*_max_ increase, which is usually strongly desired.

**Figure 1:**
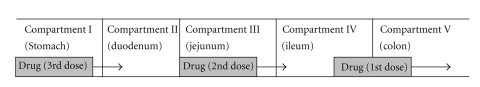
Tandem dose scheme.

In our previous study, an effective tandem dose delivery method was successfully established [12]. This novel dose strategy is based on animal anatomy and biological rhythms. The theory was focused on utilizing animal gastrointestinal (GI) transit time and considered the gastrointestinal track to be a multicompartment system. In a one-direction multicompartment GI model as illustrated in Figure 1, the stomach and small intestine (duodenum, jejunum, and ileum) were considered to be the major compartments of the system and each compartment was considered to be acting as an individual unit. In this model, when drug is dosed, the excretion from stomach to small intestine is considered to start immediately. The geometric center of the un-dissolved drug mass moves along from compartment to compartment and eventually reaches the large intestine. Little absorption takes place in the large intestine, and so it is not considered an active compartment in this model. The model is based on treating the drug mass as one band, and the geometric center of that band is located at the point of the highest density of un-dissolved drug. Once the geometric center of the dose passes through a compartment, the previous compartment is considered empty and ready for another round of drug. If we utilize this compartment again as soon as it becomes available and space the doses correctly, we should be able to use a more frequent dose in a short time frame thus approximating “oral infusion." Several researches have reported the GI transit time of small lab animals [13–22]. Based on those reported values and in-house data, the GI transit time for a rat is anywhere from 2.5 hrs to 12 hrs. The previously tandem dose work we have done used a fixed dose interval of 2.5 hrs as a starting interval to test the theory. It is believed that an interval of two to three hours should be sufficient to separate two doses from each compartment. Thus, an absorbable amount of drug can be dosed every two to three hours as a tandem dose without having significant dose overlap. This tandem dose approach provided several advantages compared with regular b.i.d. or t.i.d. doses. First, this approach eliminates the need for overtime and late night shifts. Second, unlike regular b.i.d. or t.i.d. doses that often only improve AUC for drugs with higher clearance, this approach allows for continuous absorption of drug. This allows the drug concentration in plasma to build up via accumulation, resulting in a much higher *C*_max_ which is critical for target proof of concept (POC) and safety evaluation.

**Figure 2:**
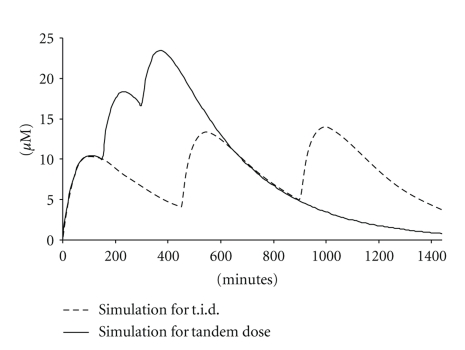
PK simulation of tandem versus regular t.i.d. dose.

The impact on AUC and *C*_max_ of a hypothetical compound by a 3X tandem dose with a 2.5 hrs interval versus that of a t.i.d. dose is illustrated in Figure 2. The PK parameters used for the hypothetical compound are representative of several internal preclinical candidates. The compound is assumed to have an oral bioavailability of 30% with a volume of distribution (Vd) of 1 L/Kg and medium clearance (CL) in rat of 20 ml/min/Kg. A previously established in-house oral model based on the Bateman equation was used for the simulation [12]. This approach has been proven to be very effective in the preclinical setting. We have demonstrated that with this oral tandem dose, higher exposures (*C*_max_ and AUC) are achievable without employing enabling formulations and while conserving the amount of active pharmaceutical ingredient required [12]. Most importantly, no extra staffing resources were needed.

Despite the success of this GI transit time-based tandem dosing strategy, one question remained. The optimum tandem dose interval had yet to be fully studied. A fixed 2.5 hrs dosing interval was used in the previous study and successfully demonstrated the theory. However, in order to take full advantage of this novel strategy, a better understanding of dose versus interval was needed. In both studies, a low solubility compound was tested with tandem dose. Compound 1 is a potent phosphodiesterase 2 (PDE2) inhibitor. PDE2 is one of the most important downstream targets of phosphodiesterase. It has been reported that inhibition of PDE2 will result in the regulation of cyclic guanosine monophosphate (cGMP) signaling and may affect anxiety-related behavior through reduction of oxidative stress [23–26]. This compound was found to have good potency with an IC_50_ value of less than 5 nM in the *in vitro* assay and good permeability by Caco-2 assay. However, the physical properties of the free form of Compound 1 were not suited for dose escalation to deliver the desired exposure. Compound 1 was highly crystalline, and the solubility of the crystalline free base was approximately 10 *μ*M in pH 6.5 buffer. This suggested that at higher doses, oral absorption of Compound 1 would most likely be solubility limited (BCS class II). 

 Based on the earlier single-dose exposure data, the upper limit of the linear dose range of Compound 1 was found to be 300 mg/Kg [12]. A much improved exposure (compared with s.i.d.) was observed when compound 1 was tandem dosed using an interval of 2.5 hrs. In this study, we further compared the impact on exposures by altering both dose amount and dose interval. Our data demonstrates that optimizing dosing interval based on dose amount can significantly increase *in vivo* exposure. Our effort has demonstrated the validity and practicality of the novel tandem dose for preclinical drug delivery. 

## 2. Materials and Methods

### 2.1. Materials

HPLC grade acetonitrile was obtained from Burdick & Jackson (Muskegon, MI) and reagent grade formic acid, sodium hydroxide obtained from EM Science (Gibbstown, NJ). The HPLC system used was an Agilent HP 1100 HPLC equipped with a diode array (DAD), a variable wavelength UV (VWD) detector, and a quaternary solvent delivery system (Palo Alto, CA). The LC/MS system used a Shimadzu solvent delivery system and a CTC PAL autosampler combined with a SCIEX 4000 tandem mass spectrometer from Applied Biosystems (Foster City, CA). A Zorbax SB-C8 column (5 *μ*m, 4.6∗150 mm) was selected and used for HPLC analysis, and a Thermosil Aquasil C18 column (3.5 *μ*m, 2.1∗50 mm) was used for LC/MS. For HPLC analysis, the water purification system used was a Millipore milli-Q system. For LC/MS, HPLC grade water from EMD Scientific, Inc. was used.

Powder X-ray diffraction (PXRD) was done on either a Bruker D-8 Advance diffractometer or a Bruker D-8 Discover with GADDS diffractometer. In both cases, Cu ka radiation was employed. For the D-8 Advanced, in-house fabricated aluminum inserts or inserts with a Hasteloy sintered filter (0.45 *μ*m) pressed in the center and held in Bruker plastic sample cup holders were utilized for all analyses. A Beckman Coulter (Miami, FL) LS 230 particle size analyzer using the small volume accessory was employed for analyzing particle size. Particle size distribution was computed by the software using Mie scattering theory, and a PIDS obscuration water optical model was employed.

Compound 1 was prepared at Pfizer, and materials used for all *in vitro* and *in vivo* studies were from the same preparation. All other chemicals were obtained from Sigma-Aldrich (St. Louis, MO) and were used without further purification.

### 2.2. Solubility, Solid-State Properties, and Formulation Evaluation of the Free Base

The solubility of Compound 1 was assessed by stirring a small amount of crystalline free base in scintillation vials that contained 5 mL of various pH buffers and FASSIF (fasted state simulated intestinal fluid). Samples were checked periodically to ensure that they were saturated with excess solid. At the end of 48 hrs, a final pH reading was taken for each sample and a representative amount of the slurry was aliquoted into centrifuge tubes. These were centrifuged at 14,000 rpm for a period of two hours. Supernatants were transferred into individual HPLC vials, and the concentration was determined by HPLC (DAD). The remaining solid form was analyzed by PXRD. Formulations with aqueous media were prepared by suspending bulk drug in a vehicle containing 0.5% Methylcellulose and 0.1% Tween 80 in distilled water. Formulation concentrations were adjusted to dose with a fixed dosing volume for all doses (total dose 20 mL/Kg/day). Particle size distribution of each formulation was determined on a Beckman Coulter LS 230 particle size analyzer. 

#### 2.2.1. In Vivo Methodology

For *in vivo* work, male Sprague-Dawley (SD) rats were purchased from Charles River Laboratories (Wilmington, MA). This animal study was approved by the St. Louis Pfizer Institutional Animal Care and Use Committee. The animal care and use program is fully accredited by the Association for Assessment and Accreditation of Laboratory Animal Care, International. All oral doses other than standard Pharmacokinetic studies were performed under “fed" condition to better estimate the multiday toxicology study. The oral dose volume was based on 20 mL/Kg/day of body weight for all studies. All doses were based on mg/Kg of body weight. Rats were catheterized in the jugular vein and carotid artery for iv dosing and sampling, respectively. At each time point, 150 uL of blood was withdrawn from each animal, and replaced by saline. Blood sampling was carried out using a Culex Automated Blood Sampling System (West Lafayette, IN) and collected in microtainer plasma separator tubes with lithium heparin using heparinized capillary tubes. Plasma samples were obtained by centrifugation at 8000 rpm for 10 minutes, and 20 *μ*L of the plasma sample was extracted with 180 *μ*L of acetonitrile containing 0.25 *μ*M of the internal standard (prepared in house). The precipitated samples were centrifuged, and supernatant was transferred to a 96-well plate. Analytical standards were prepared by spiking known amount of standards into control plasma and followed the above extraction procedure. Tandem dosing (three times) was performed at 50, 100, and 200 mg/kg and dose intervals were 1, 1.5, and 2.5 hrs.

Plasma samples were analyzed by LC/MS/MS. A Shimadzu LC (LC 20 AD) multiple solvent pump system was used for the gradient elution. A CTC Pal autosampler was used for sample injection. Solvent line A contained HPLC grade water with 0.1% formic acid (v/v), and Solvent line B contained acetonitrile with 0.1% formic acid (v/v). The flow rate was set at 0.4 mL/minute, and a Thermo Aquasil C18 20 × 2.1 mm, 3.5 micron column was used for analysis. At T(0), the mobile phase (90% A and 10% B) was mixed by the HPLC pump and held for 0.5 minutes (isocratic elution). From T(0.5) to T(1.5) minutes, a linear gradient from 10% B to 90% B was applied and allowed to hold at 90% B for 1 minute (from 1.5 to 2.5 minutes). At T (2.7) minutes, the system was set back to the initial condition allowed to equilibrate for 1.3 minute to prepare for the next injection. The analyte was quantified versus a plasma standard curve using a Sciex API 4000 mass spectrometer with an internal standard. For the analysis, positive electrospray mode was used. For sample preparation in general, 20 *μ*L plasma was extracted with 180 *μ*L acetonitrile containing 0.25 *μ*M of the internal standard carbamazepine.

#### 2.2.2. Modeling

Pharmacokinetic analysis was performed using Watson 7.2 Bioanalytical LIMS system by Thermo Electron Corporation (Thermo Fisher Scientific, Waltham, MA). An in-house model based on the Bateman equation was used for the simulation

(1) $$\text{Cp}\left(t\right)=\frac{\left(Ka{*}^{}F{*}^{}Dpo{*}^{}\left(e^{-Kt}-e^{-Kat}\right)\right)}{V\left(Ka-K\right)}.$$

Cp(*t*): plasma concentration as a function of time. *Ka*: absorption rate constant. *K*: elimination rate constant. *F*: bioavailability. *Dpo*: dose (oral). *V*: volume of distribution. *t*: time

The Wagner-Nelson equation was used to calculate drug absorbed to further assess the absorption as a function of time.

(2) $$\begin{array}{cc} dA & =V{*}^{}d\text{Cp}+V{*}^{}k{*}^{}\text{Cp}{*}^{}dt,\\ A & =V{*}^{}\text{Cp}+V{*}^{}K{*}^{}\overset{t}{\underset{0}{\int }}\text{Cp}{*}^{}dt, \end{array}$$

where *A* = drug absorbed. *V* = volume of distribution. C_p_ = plasma concentration. *K* = elimination rate constant. *t* = time. *Fraction absorbed = ( BA ∗ Hepatic Blood Flow)/(Hepatic Blood Flow-Clearance). Rat Hepatic Blood Flow is ∼ 70 *mL/min/kg. Absorption rate constant *Ka* = 1/((MRT)_po_ − (MRT)_iv_)). *T*_1/2abs_ = ln 2/*Ka*.

## 3. Results and Discussions

Basic pharmacokinetic parameters of Compound 1 were obtained from low dose IV (1 mg/Kg) and oral (3 mg/Kg) experiments in rats (*n* = 3). Compound 1 was found to have medium CL, a Vd of 6 L/kg, and an oral bioavailability of 60%. The absorption constants (*Ka*) for both compounds were calculated using the mean resident time (MRT) method by assuming the absorption of Compound 1 followed the single first-order kinetic process [27]. The absorption half-life was calculated to be approximately 0.87 hr for Compound 1. Additional PK and physicochemical information of Compound 1 is listed in Table 1. The fraction absorbed was calculated by assuming that CL was mainly hepatic. The fraction absorbed (FA) was calculated to be approximately 0.79. 

For Compound 1, oral absorption was not an issue when doses were low. However, good FA at low doses does not always translate to good FA when the dose is increased. Given the low solubility of the compound, solubility limited absorption is still likely to occur at higher doses (BCS II). This presented a major hurdle as Compound 1 advanced to safety studies where high exposures (both *C*_max_ and AUC) were needed. In the first single dose range finding safety study, Compound 1 was dosed in rats as suspension formulations. Compound 1 was dosed once a day (s.i.d.) at 300, 600, and 1000 mg/kg in rats. Nondose proportional AUC and *C*_max_ increases were observed (see Table 2).The exposure increase between 300 mg/Kg and 600 mg/Kg doses was small. A two-fold dose increase only resulted in a 0.2 times increase in AUC and a 0.39 times increase in *C*_max_. Even smaller increments were found when comparing exposures between the 600 mg/Kg dose and 1000 mg/Kg dose. Exposure (especially the *C*_max_) was not high enough to establish appropriate margins to assess the safety liabilities. The exposures for Compound I reached a plateau at high doses. As compound 1 possesses low aqueous solubility, it was hypothesized that for Compound 1, absorption was limited by solubility at high dose. Formulation options were evaluated for Compound 1 in order to improve the exposure. *In vitro* data (not included) led us to believe that improving exposure sufficiently via formulation would be time consuming, expensive, and therefore not an option. Regular multidoses b.i.d. (every 12 hrs) or t.i.d. (every 8 hrs) were considered and found less favorable since increased staffing and overtime pay (for late night dosing) would be required. Upon close examination of the data, Compound 1 exhibits nondose dependent exposure increases at higher doses. However, it is entirely possible that oral absorption may be linear at doses below the lowest dose (300 mg/Kg). Based on this assumption, we hypothesized that if each dose is less than 300 mg/Kg and administered in a tandem dose scheme, the high FA (for each dose) and short dose frequency (every 2.5 hrs) would allow drug exposure to build up very quickly (both AUC and *C*_max_). The 2.5 hrs dose interval was chosen to test at first [12]. The 2.5 hrs interval was picked based on the literature reviews [12–22] and in house data (not included). This dose interval has successfully demonstrated to be sufficient to separate two doses (represented by the geometric mean of the unabsorbed drug at the moment) from each compartment [12]. In general, rats were dosed at 7 o'clock in the morning (first dose), nine thirty (second dose), and twelve o'clock (third dose). The exposure results obtained from the tandem dose were very encouraging. In general, much higher *C*_max_ and AUC were obtained by the tandem dose compared with the s.i.d. dose. The 100 mg/Kg tandem dose (300 mg/kg total) gave an AUC and *C*_max_ similar to the 1000 mg/Kg s.i.d. dose. The 200 mg/Kg tandem dose (600 mg/kg total) resulted in double the exposure of the 1000 mg/Kg s.i.d. dose. Most importantly, the *C*_max_ increase was observed as predicted. Thus, the utility of this novel tandem dose method has been demonstrated by lowering the total drug requirement and still achieving significantly improved exposures for low solubility compounds [12]. A pharmacokinetic model was established in house by using the Bateman equation (assuming linear pharmacokinetics) to estimate the exposure from a tandem dosing scheme with success [12].

Despite the success of the tandem dose approach, one key question remained: what is the optimum dose interval for a given dose? It is understood that when dose increases, so does the amount of drug remaining in the GI The risk of drug “overlap” in the GI may increase when the tandem dose interval is shortened. When this “overlapped” portion becomes significant, the fraction of nonabsorbable drug will increase and result in lower exposure even for a tandem dose (similar to high s.i.d. dose). Vice versa, when a lower dose is given, the amount of drug remaining in the GI is reduced and drug overlap from a tandem dose scheme (i.e., 2.5 hrs intervals) is less likely. Thus, a shorter interval could be used and may provide better efficiency. For this study, three different dose levels (50, 100, and 200 mg/Kg X3 tandem) were used alone with three different dose intervals (1, 1.5, and 2.5 hrs). A detailed dose scheme is listed as Table 3. The overall goal is to further study and optimize the tandem dosing scheme.

**Figure 3:**
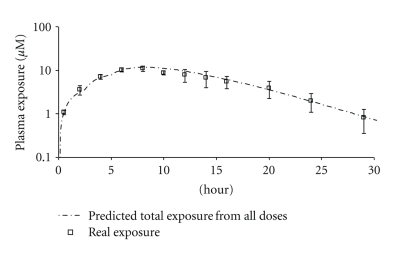
Compound 1 50 mg/Kg X3 tandem dose (2.5 hr interval) predicted versus observed exposure.

**Figure 4:**
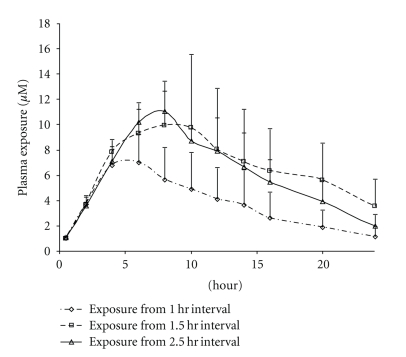
Compound 1 50 mg/Kg X3 tandem dose (1, 1.5, and 2.5 hrs interval) exposure comparison.

**Figure 5:**
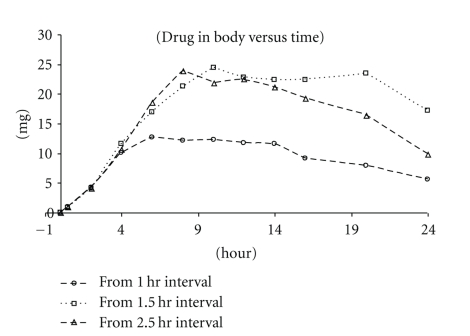
The 50 mg/kg X3 Tandem Dose Wagner-Nelson Plot (presented as mean values).

All doses were successful and well tolerated by the animals. For the 50 mg/Kg X3 tandem dose, the best efficiency was found when the 1.5 and 2.5 hr intervals were used. The higher Cmax and AUC obtained via the tandem doses were well within our model prediction (an example is presented as Figure 3). The exposures obtained by this 50 mg/Kg X3 tandem dose are comparable to 300 mg/kg s.i.d dose, and only half the amount of drug was used. The shortest interval (1 hr) was found to be the least effective and delivered the lowest *C*_max_ and AUC; however, it was still respectable. It is hypothesized that with such a short interval, drug “overlapped” from dose to dose, increasing the nonabsorbable portion and thereby reducing the exposure (similar to that of an s.i.d. dose). Better drug delivery efficiency was achieved when the dose interval was increased to 1.5 and 2.5 hrs. *C*_max_ and AUC from both dosing schemes were comparable. This suggests that for this (low) dose, 1.5 hrs was sufficient to physically separate the doses in the GI Exposure profiles of the 50 mg/Kg tandem dose are presented in Figure 3. The effects of tandem dosing were very clear when comparing the absorption phases (*α* phase) of the three dosing curves (Figure 4). With all three intervals, the absorption phases (rate of uptake) were very similar and the AUC/Dose (for 1.5 hr interval) was calculated to be 1.06 ± 0.46 *μ*M*hr/mg/kg. The effect of the tandem dose is made evident by the longer absorption phase generated by both the 1.5 and 2.5 hrs dosing intervals. The Wagner-Nelson equation (see Section 2) was used to calculate the drug absorbed and further assess the absorption as a function of time. According to the calculation, the *δA*/*δT* value for all three dosing schemes was approximately 2 mg/hr. The higher exposures observed from the longer dosing interval were attributed to the increased absorption time (Figure 5).

**Figure 6:**
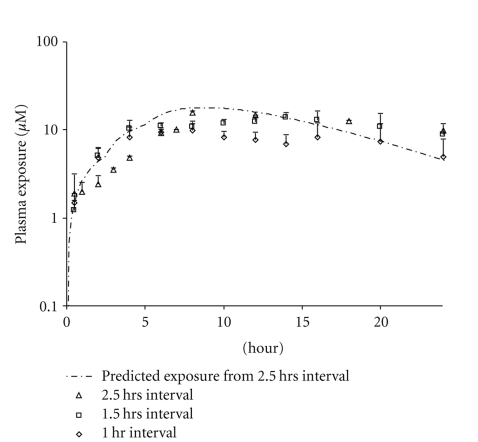
The 100 mg/kg X3 Tandem Dose Predicted (2.5 hr) versus Obtained Exposures from 1, 1.5, and 2.5 hrs interval.

**Figure 7:**
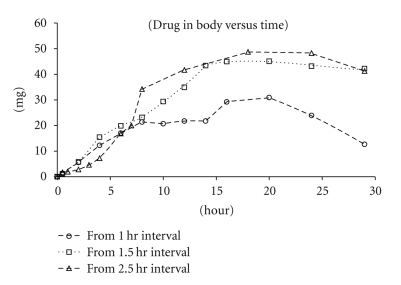
The 100 mg/kg X3 Tandem Dose Wagner-Nelson Plot (presented as mean values).

 For the 100 mg/Kg tandem dosing groups, similar impacts were observed when the dose interval changed. In general, the shortest dosing interval (1 hr) gave the lowest exposures. Again, it is hypothesized with such a short interval, drug “overlapped” from dose to dose which caused the nonabsorbable portion to increase thereby reducing the exposure (similar to an s.i.d. dose). Better drug delivery efficiency was achieved when the dose interval increased to 1.5 and 2.5 hrs. The *C*_max_ and AUC (Tables 4 and 5) obtained from both dosing schemes are comparable and well exceed the values from the s.i.d. dose (Table 2 300 mg/kg). This suggests that for this dose (100 mg/Kg X3) 1.5 hrs may be sufficient to separate two doses as well. However, it is worth noticing that the variability of data obtained from the 1.5 hr interval is higher than that of the 2.5 hr interval. This suggests that the 1.5 hr interval may not be ideal for higher doses as the risk of drug overlap in the GI is higher and may have contributed to the higher variability in exposures. The simulated exposure (2.5 hrs interval) versus the obtained exposure for the 100 mg/kg X3 tandem dose is presented in Figure 6, and the AUC/Dose (for 2.5 hr interval) was calculated to be 1.03±0.05  *μ*M*hr/mg/kg. Based on the linear model and exposures obtained from the 1.5 and 2.5 hr intervals, a noticeably increased beta phase half-life was observed from the tandem doses versus the predicted curve. It is possible that via accumulation the drug exposure has reached the nonlinear range (saturated the CL), and therefore a linear PK model underpredicts the beta phase half-life. A Wagner-Nelson plot (see Section 2) was used to calculate drug absorbed and to assess the absorption as a function of time and is presented as Figure 7. Again, the higher exposures observed from the longer dosing interval were attributed to increased absorption time. 

**Figure 8:**
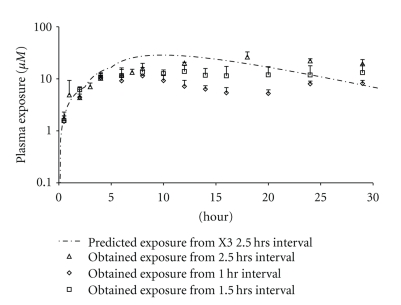
The 200 mg/kg X3 Tandem dose predicted (2.5 hr) versus obtained exposures from 1, 1.5, and 2.5 hrs interval.

**Figure 9:**
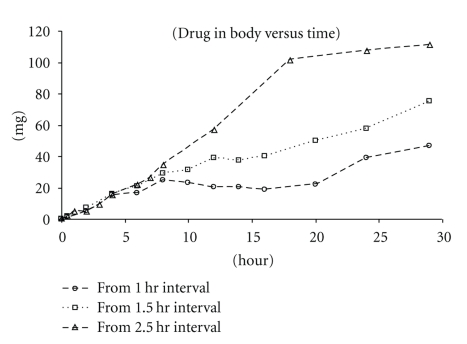
The 200 mg/kg X3 Tandem Dose Wagner-Nelson Plot (presented as mean values).

For the 200 mg/Kg tandem dosing groups, a much bigger impact was observed when dose interval changed. In general, the shortest dosing interval (1 hr) gave the lowest exposure followed by the 1.5 hr interval. Again, it is hypothesized that with such a short interval, drug “overlapped” from dose to dose which caused the nonabsorbable portion to increase thereby reducing the exposure (similar to a s.i.d. dose). Better efficiency was achieved when the dose interval increased to 2.5 hrs. Better drug delivery efficiency was achieved when the dose interval increased to 1.5 and 2.5 hrs. This is not surprising, since for a low solubility drug, the nonabsorbable portion increases when the dose increases due to solubility limited absorption. It is also understood that the dose interval should be Tmax (from single dose) dependent. The overlap from the shorter dosing interval becomes more significant and the nonabsorbable portion increases as dose increases; thus, the dosing interval and the dose are interdependent, and both must be considered in order to minimize the “drug overlap" in the GI track. The *C*_max_ and AUC (Tables 4 and 5) obtained from the 1.5 hrs dosing scheme are comparable to the values obtained from the s.i.d. dose (Table 2. 1000 mg/kg). *C*_max_ and AUC from the 2.5 hrs dosing scheme well exceed the values obtained from the s.i.d. dose of 1000 mg/kg. The obtained *C*_max_ of 200 mg/kg tandem dose (with 2.5 hr interval) was 26.3 ± 4.0 *μ*M, and AUC/Dose (for 2.5 hr interval) was calculated to be 0.87 ± 0.08 *μ*M*hr/mg/kg.This suggests that for this dose (100 mg/Kg X3) 1.5 hrs may be sufficient to separate two doses; however, the 2.5 hr interval delivers the best results. Similarly, the data obtained from the 1.5 hr interval exhibits higher variability when compared to the 2.5 hr interval. This again suggests that 1.5 hrs may not be the ideal interval for higher doses as the risk of drug overlap in the GI is higher and may contribute to higher variability in exposures. The variability could also be subject dependent. The simulated exposure (2.5 hrs interval) versus obtained exposure for 200 mg/kg X3 tandem dose is presented in Figure 8. A Wagner-Nelson plot (see Section 2) was used to calculate drug absorbed and to assess the absorption as a function of time for the 200 mg/kg X3 dose is presented as Figure 9. Similarly, a noticeable increase in beta phase half-life was observed for the tandem doses versus the predicted curve using our linear PK model. It is possible that via accumulation drug exposure has reached the nonlinear range (saturated the CL), and therefore a linear PK model underpredicts the beta phase half-life. It is also possible that this phenomena was due to the larger amount of drug dosed which altered some physiological factor (i.e., transit time) in the animal or saturated the absorption. Thus, the parameters generated by simple PK studies at a lower dose may not be sufficient to predict every aspect of a higher dose study. However, since both AUC and *C*_max_ were actually well within our target, we believed this model work well. It is also hypothesized that when other factors are kept constant and compound solubility is improved, the shorter interval may work better. Better solubility will allow for faster absorption of drug, less will remain in the GI, and drug overlap will no longer be an issue.

The above data strongly support the tandem dose approach to increase exposure while minimizing compound usage. The present work supports the transit time theory in rats. We have also demonstrated that the ideal interval is dose dependent. In summary, significantly improved exposures were obtained by using the tandem dose with the appropriate interval. A simple calculation of dose efficiency was performed based on using 40% less drug (600 mg/kg versus 1000 mg/kg) and doubling the exposure. This tandem dose has improved the dose efficiency by approximately 300% for Compound 1. This conservative calculation was done by assuming a linear increase of both *C*_max_ and AUC from 1000 to 2000 mg/Kg doses for both compounds. This assumption is an overestimation since exposure increases of Compound 1 (s.i.d) were proven nonlinear beyond 300 mg/Kg (and the actual nonlinear dose could be lower than 300 mg/Kg). Thus, the true efficiency could be much higher. This novel tandem dose oral delivery approach using an optimized dosing interval achieves significantly higher *in vivo* exposure using less drug and requires no additional resources. It is simple, cost effective, and well tolerated by animals and should be further utilized in industry. Regular b.i.d. or t.i.d. doses take up to 12 or 16 hours to administer. Depending on the dose, a simple X3 tandem dose can be administered within 2–5 hrs (1 to 2.5 hr interval). This easily fits into the traditional work day, and no additional staff or overtime is necessary. In theory, the tandem dose is not limited to three doses per day; a fourth dose can be given to further boost the exposure if needed without altering the normal eight-hour work day [12]. Our current investigation of dosing interval further refines the tandem dosing strategy. This improved strategy can positively impact the preclinical oral delivery of low solubility compounds.

## 4. Conclusion

In our research, we utilized this novel tandem dose strategy in rat and assessed the impact of dosing intervals on exposure. We successfully demonstrated that by using the tandem dose strategy with the appropriate dosing interval, significantly higher *in vivo* exposure can be reached without extraresources and investments. This method is well tolerated by the animal and achieves increased exposure with less drugs dosed. This novel approach allows the preclinical researcher to quickly evaluate the *in vivo* efficacy and safety of a new target. We believe that by using an approach similar to the system described above, reliable data for decision making can be obtained earlier in the discovery process prior to the need for substantial investments.

## Figures and Tables

**Table 1:** Basic PK and physicochemical parameters of compound 1.

Parameters	Compound 1
*Ka* (−1/hr)	0.8
CL (mL/min/kg)	22
*V*dss (L/kg)	6.0
*T*_1/2_ (Hrs)	6.9
MRT Oral (3 mg/kg)/IV (1 mg/kg)	6.1/4.8 (hrs)
Oral BA (3 mg/kg)	60%
Aqueous solubility (pH 6.5)	10 *μ*M
Solubility in FASSIF	15 *μ*M
pKa (between pH 2 and 10)	None
Log *D* (pH 7.4)	3.0

**Table 2:** Exposure of compound 2 from s.i.d. dose.

Dose (mg/kg) (*N* ≥ 3)	Frequency	*C*_max_ (*μ*M)	AUC (*μ*M*hr)	AUC/Dose ± SD (*μ*M∗hr/mg/kg)
300	Once a day	10.6 ± 2.8	167 ± 36	0.565 ± 0.120
600	Once a day	12.9 ± 1.2	232 ± 29	0.387 ± 0.049
1000	Once a day	14.4 ± 2.2	273 ± 66	0.273 ± 0.066

**Table 3:** Detailed tandem dose scheme and grouping (*n* ≥ 3 for each group).

Dose (mg/Kg)	Frequency/total dose	1 hr interval	1.5 hrs interval	2.5 hrs interval
50 mg/Kg	X3 tandem (150 mg/Kg)	Group 1	Group 2	Group 3
100 mg/Kg	X3 tandem (300 mg/Kg)	Group 4	Group 5	Group 6
200 mg/Kg	X3 tandem (600 mg/Kg)	Group 7	Group 8	Group 9

**Table 4:** Tandem dose scheme AUC comparison (*μ*M*hr).

Dose (mg/Kg)/Interval (hrs)	1 hr	1.5 hr	2.5 hr
50 (mg/Kg) X3/AUC/Dose	85 ± 41	159 ± 69	147 ± 31
100 (mg/Kg) X3	192 ± 58	289 ± 55	310 ± 14
200 (mg/Kg) X3	210 ± 26	330 ± 90	521 ± 47

**Table 5:** Tandem dose scheme average *C*_max_ comparison (*μ*M).

Dose (mg/Kg)/Interval (hrs)	1 hr	1.5 hr	2.5 hr
50 mg/Kg X3	7.2 ± 2.0	10.8 ± 4.9	11.1 ± 1.6
100 mg/Kg X3	11.1 ± 2.1	14.7 ± 2.5	15.9 ± 0.4
200 mg/Kg X3	12.0 ± 0.4	17.0 ± 3.7	26.3 ± 4.0
